# Coculture with astrocytes reduces the radiosensitivity of glioblastoma stem-like cells and identifies additional targets for radiosensitization

**DOI:** 10.1002/cam4.510

**Published:** 2015-10-30

**Authors:** Barbara H Rath, Amy Wahba, Kevin Camphausen, Philip J Tofilon

**Affiliations:** Radiation Oncology Branch, National Cancer InstituteBethesda, Maryland, 20892

**Keywords:** Astrocytes, glioblastoma stem-like cells, microenvironment, radiosensitization, STAT3

## Abstract

Toward developing a model system for investigating the role of the microenvironment in the radioresistance of glioblastoma (GBM), human glioblastoma stem-like cells (GSCs) were grown in coculture with human astrocytes. Using a trans-well assay, survival analyses showed that astrocytes significantly decreased the radiosensitivity of GSCs compared to standard culture conditions. In addition, when irradiated in coculture, the initial level of radiation-induced *γ*H2AX foci in GSCs was reduced and foci dispersal was enhanced suggesting that the presence of astrocytes influenced the induction and repair of DNA double-strand breaks. These data indicate that astrocytes can decrease the radiosensitivity of GSCs in vitro via a paracrine-based mechanism and further support a role for the microenvironment as a determinant of GBM radioresponse. Chemokine profiling of coculture media identified a number of bioactive molecules not present under standard culture conditions. The gene expression profiles of GSCs grown in coculture were significantly different as compared to GSCs grown alone. These analyses were consistent with an astrocyte-mediated modification in GSC phenotype and, moreover, suggested a number of potential targets for GSC radiosensitization that were unique to coculture conditions. Along these lines, STAT3 was activated in GSCs grown with astrocytes; the JAK/STAT3 inhibitor WP1066 enhanced the radiosensitivity of GSCs under coculture conditions and when grown as orthotopic xenografts. Further, this coculture system may also provide an approach for identifying additional targets for GBM radiosensitization.

## Introduction

Radiotherapy is a primary treatment modality for glioblastomas (GBMs) 1. However, while many GBMs initially respond to radiation, even in combination with surgery and chemotherapy, median survival continues to be less than 2 years after diagnosis 2. GBM recurrence is typically within the initial radiation treatment volume indicating that GBM cells in situ are extremely radioresistant 3. Defining the processes and molecules responsible for this radioresistance should thus provide a rational basis for designing target-based strategies that enhance GBM radiosensitivity. Investigations into such radioresistance have traditionally focused on long established glioma cell lines, the biology of which has little in common with GBM in situ 4. With respect to a more biologically accurate model system, data now suggest that GBMs are driven and maintained by a subpopulation of clonogenic cells referred to as glioblastoma stem-like cells (GSCs). GSCs have a number of in vitro properties in common with normal neural stem cells 5–7 and brain tumor xenografts initiated from GSCs simulate the genotype and gene expression patterns of the GBM from which they originated 8.

As a model system for investigating GBM radioresistance, we previously determined the in vitro radiosensitivity of GSCs using a clonogenic assay, the gold standard for defining intrinsic radiosensitivity. Results showed that, whereas there was variability among the GSCs, each of the GSC lines evaluated were actually more radiosensitive than established glioma cell lines 9. Thus, the applicability of GSCs as an in vitro model for investigating the mechanisms mediating GBM radioresistance was unclear. However, these initial in vitro studies failed to account for the normal brain milieu. As an initial investigation into the role of the microenvironment in GBM radioresistance, *γ*H2AX and 53BP1 nuclear foci were used to compare the radioresponse of GSCs in vitro to that of cells within GSC-initiated intracerebral (ic) xenografts 10,11. These nuclear foci provide a measure of radiation-induced DNA double-strand breaks (DSBs) 12–14, the critical lesion in radiation-induced cell death, and allow for the direct comparison of cells under in vitro and in vivo growth conditions. In this model system, the initial level of radiation-induced foci was significantly reduced in tumor cells within ic xenografts and the foci that did form dispersed more rapidly as compared to cells irradiated under the in vitro conditions. These results suggested that GSCs grown orthotopically are less susceptible to DSB induction and have an increased capacity to repair DSBs and thus are less radiosensitive than cells grown in vitro. Moreover, these data implied that to define the mechanisms mediating GBM radioresistance it will be necessary to take into account the microenvironment.

Toward this end, we have extended our in vivo studies to an in vitro model involving the coculture of GSCs and normal human astrocytes. While the brain consists of a number of phenotypes, astrocytes are the most abundant non-neural cell type comprising ∼50% of the human brain volume. Astrocytes are a primary source of growth factors, cytokines, and other bioactive molecules 15,16 and play a critical role in the brain response to multiple types of injury 17,18. The data presented here show that the presence of astrocytes in the coculture model decreases the radiosensitivity of a panel of GSCs, an effect mediated through a paracrine mechanism. In addition, a protein (STAT3) activated under coculture conditions, is shown to provide a target for GBM radiosensitization. These results are consistent with the brain microenvironment contributing to GBM radioresistance in situ.

## Materials and Methods

### Cell lines and treatment

The neurosphere-forming cultures NSC11, NSC23 (kindly provided by Dr. Frederick Lang, MD Anderson Cancer Center), GBAM1 9, and 923 19 were isolated from human GBM surgical specimens as described previously 6. Neurospheres were maintained in stem cell medium (DMEM/F12, B27 [Thermo Fisher Scientific, Grand Island, NY]) and basic fibroblast growth factor (bFGF), epidermal growth factor (EGF) (50 ng/mL each, R&D Systems, Minneapolis, MN) at 37°C, 5%CO_2_/5%O_2_. CD133+ or CD15+ cells were isolated from the specified GBM neurosphere cultures by fluorescence-activated cell sorting (FACS) as reported previously 9. All CD133+ and CD15+ cell cultures met the criteria for GSCs. For in vitro experiments, GSCs were disaggregated and seeded into poly-l-ornithine/laminin (po/ln; Sigma-Aldrich, St. Louis, MO)-coated cell culture dishes. Under these conditions GSCs grow as an adherent monolayer maintaining their respective surface marker expression and stem-like characteristics 20. Human astrocytes (ScienCell, Carlsbad, CA) were cultured according to company’s protocol; U251 glioma cells were obtained from ATCC. All cells were cultured less than 3 months after resuscitation. WP1066 (Selleckchem, Houston, TX) was dissolved in dimethyl sulfoxide (DMSO) for in vitro experiments. Cell cultures were irradiated with doses up to 8 Gy using 320 kV X-ray source at 2.3 Gy/min (Precision XRay Inc., North Branford, CT).

### Coculture radiation survival assay

Single cell suspensions of tumor cells were seeded onto po/ln-coated multiwell plates for monoculture (control) and coculture, respectively (NSC11 and GBAM1 at 500 cells/cm^2^; NSC23 and 923 at 250 cells/cm^2^). For coculture experiments, astrocytes (1.33 × 10^6^ cells/cm^2^) were seeded onto po/ln-coated trans-well inserts (GreinerBioOne, Monroe, NC, 0.4 *μ*m pore size); after attachment (overnight) in astrocyte medium (ScienCell) the inserts were washed twice with stem cell medium and transferred to multiwell plates carrying GSCs. Single dose of radiation (2–8 Gy) was delivered using a 320 kV X-ray source at 2.3 Gy/min (Precision XRay Inc.) 48 h after coculture initiation to monoculture and coculture of GSCs. Media was changed 2 days after irradiation for all culture conditions and then every other day with tumor cell number defined after 13 days in mono- and coculture according to ATP content (CellTiter-Glow; Promega, Madison, WI) and radiation survival curves were generated as described previously 21. The same procedure was performed on U251 cells (25 cells/cm^2^) grown in DMEM/10%, fetal bovine serum (FBS).

### Immunofluorescent analysis of *γ*H2AX foci

GSCs grown alone (monoculture) or with astrocytes (coculture) were exposed to a single dose of radiation (1–6 Gy) and evaluated for nuclear *γ*H2AX foci levels as described previously 9 with the number of foci per cell determined in at least 25 cells per condition per experiment.

### Cytokine analyses

Cytokine levels were analyzed in conditioned media from astrocytes, mono-, and cocultures using the (RayBiotech, Norcross, GA) Human Cytokine Antibody Array G Series 5 according to the manufacturer’s instructions with two subarrays per sample. Data are expressed as an average of the ratio of conditioned media to control stem cell media obtained from two independent experiments.

### Gene expression analysis

For the microarray analysis, GSCs were seeded into po/ln-coated six-well plates (5 × 10^4^ cells/cm^2^) without (monoculture) or with astrocytes (1.4 × 10^6^ cells/cm^2^) onto the corresponding trans-well inserts. After allowing each cell type to adhere overnight cocultures were initiated by combining the inserts and the six-well plates; 48 h later RNA was collected from GSCs (mono- and coculture) as described previously 22. RNA (100 ng) collected from three biological replicates from each culture condition was processed and subjected to microarray analysis on Human Genome U133A 2.0 chips (Affymetrix, Santa Clara, CA) according to the manufacturer’s instructions. Using Affymetrix Expression Console, Mas5 normalization was performed on all data sets. Differentially expressed genes (cocultures vs. monocultures) were defined as *P* < 0.05 (Student’s *t*-test). The data have been deposited in NCBI’s Gene Expression Omnibus and are accessible through GEO Series accession number GSE63037. Array data were analyzed using Ingenuity Pathway Analysis (IPA) software (Qiagen Inc, Valencia, CA) for known interactions, pathway analysis, and determination of upstream regulators. Analyses were done in using the Fall 2014 IPA build.

### Immunoblot analysis

Protein from GSCs alone or after 48 h in coculture with astrocytes was collected and subjected to immunoblot analyses as described previously 22. Primary antibodies included anti-STAT3, anti-phospho-STAT3 (Y705) (Cell Signaling Technology, Danvers, MA), and anti-*β*-actin (Sigma-Aldrich). Donkey anti-rabbit and sheep anti-mouse horseradish peroxidase–conjugated secondary antibodies were purchased from GE Healthcare Life Sciences, Pittsburgh, PA.

### Orthotopic xenografts

CD133+ NSC11 cells engineered to express luciferase with the lentivirus LVpFUGW-UbC-ffLuc2-eGFP2 were orthotopically implanted into 6-week-old athymic female nude mice (nu/nu; NCI Animal Production Program, Frederick, MD); bioluminescent imaging (BLI) and local irradiation were all performed as described previously 11. WP1066 was dissolved in DMSO/Kollisolv (Sigma-Aldrich) (1:5) and delivered by oral gavage 23. At 24 days postimplantation, consistent BLI was detected in all mice, which were then randomized into four groups (9–10 mice/group) and the specified treatments initiated the next day. Mice were monitored every day until the onset of neurologic symptoms (morbidity). All experiments were performed as approved by the principles and procedures in the NIH Guide for Care and Use of Animals.

### Immmunhistochemistry

Mice bearing ic NSC11 tumors were treated at the onset of morbidity with vehicle or WP1066 40 mg/kg (oral gavage). Tumors were excised 6 h posttreatment and fixed in 10% buffered formalin. Paraffin sections were deparaffinized in xylene and rehydrated in decreasing amounts of alcohol. Sections were steamed in citrate buffer and incubated in 1% bovine serum albumin containing 10% goat serum (Sigma-Aldrich). Anti-phospho-STAT3 (Y705), anti-STAT3 (Cell Signaling), and anti-human-Nestin (EMD Millipore, Billireca, MA) were incubated overnight at 4°C followed by incubation with Alexa488- and Alexa555-labeled secondary antibody (Invitrogen) and then mounted with mounting media containing (4’,6-diamidino-2-phenylindole) DAPI; (Vector Laboratories, Burlingame, CA) to visualize nuclei. Images were generated using a Zeiss microscope (Thornwood, NY) with a 40× objective.

### Statistical analysis

GraphPad Prism 6, (GraphPad Software, La Jolla, CA) was used for all analyses. Monocultures versus cocultures were compared according to Student’s *t*-test. For in vivo survival studies, Kaplan–Meier curves were generated and log-rank values calculated.

## Results

To investigate the influence of the microenvironment on GSC radiosensitivity, a coculture model was used in which GSCs were grown in the bottom compartment of a trans-well chamber with human astrocytes seeded on the top membrane, which, while allowing for molecular diffusion, is impermeable to cell invasion. The GSCs used in this study were isolated based on the expression of CD133 (NSC11, GBAM1, NSC23) or CD15 (923) as described previously 24,25. Formerly, the radiosensitivity of GSCs had been defined by seeding cells at low density into tissue culture dishes coated with poly-l-lysine, on which GSCs form well-demarcated colonies in stem cell media allowing for clonogenic analysis 9. However, in the presence of astrocytes (i.e., coculture conditions) GSCs grow as a well-dispersed monolayer on poly-l-lysine rather than as colonies. Although consistent with astrocytes modifying the biology of GSCs 22, it eliminates the use of clonogenic analysis as a measure of radiosensitivity.

As an alternative to the clonogenic analysis of the radiosensitivity of GSCs in astrocyte coculture we used the method of Carmichael et al. 21, which allows for generation of radiation cell survival curves based on cell number. Of note, the standard approach for evaluating cytotoxicity according to cell number, often referred to as an MTT or alamar blue assay, is to determine cell number at 2–4 days after insult. Because radiation induces transient growth arrest as well as cell death, which can require multiple divisions for expression, this short-term assay does not provide an accurate representation of radiosensitivity. However, Carmichael et al. showed that extending the period between irradiation and analysis to a time sufficient for at least five cell divisions allows for the discrimination between transient arrest and cell death. Comparing directly to clonogenic survival analysis, they showed that after optimizing for cell number and allowing for a sufficient number of cell doublings after irradiation constructing survival curves based on cell number (a nonclonogenic assay) provides an accurate measure of radiosensitivity and, moreover, allows for the identification of radioresponse modifiers.

**Figure 1:**
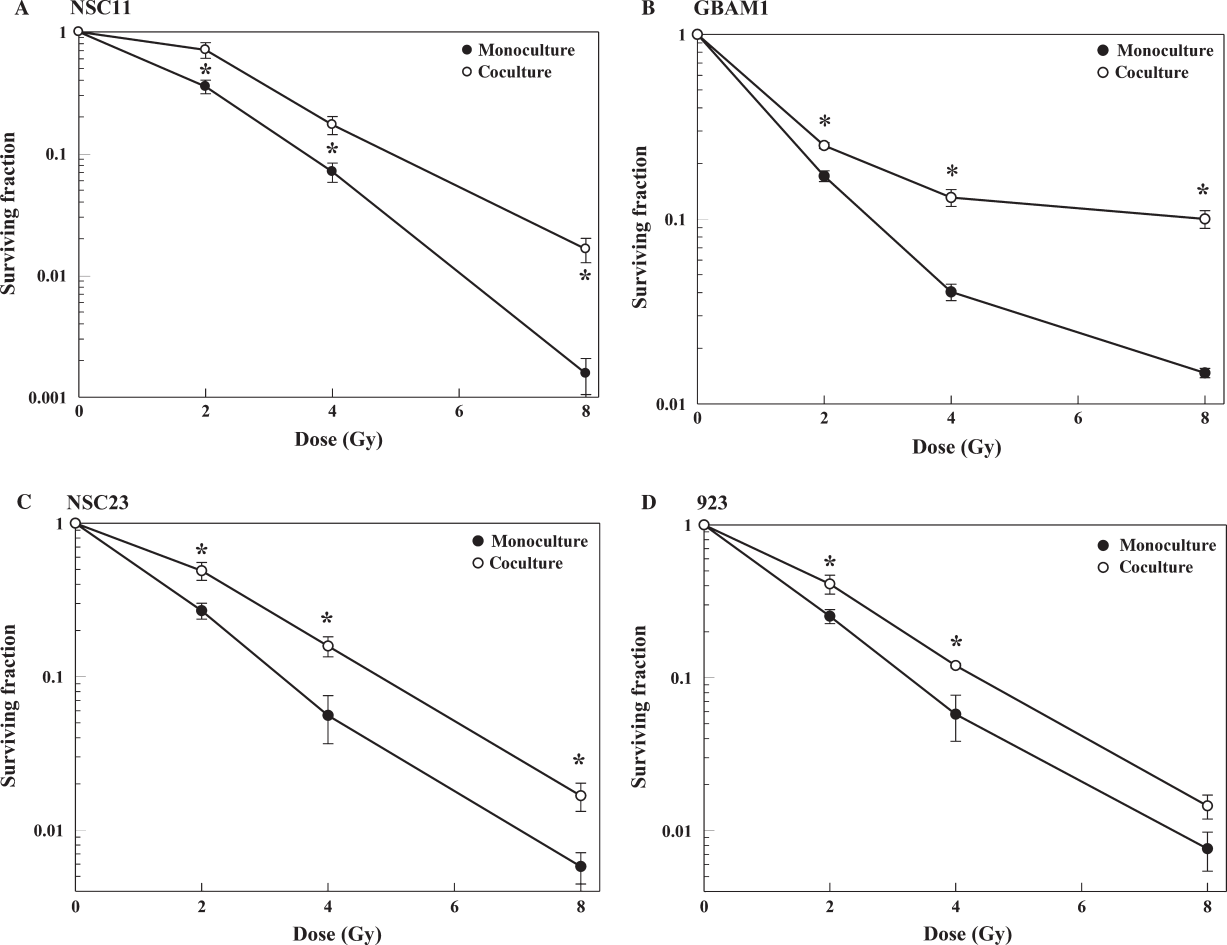
Effect of astrocytes on glioblastoma stem-like cell radiosensitivity: (A) NSC11, (B) GBAM1, (C), NSC23, (D) 923. Forty-eight hours after coculture initiation mono- and cocultures were irradiated and 11 days later cell number was determined according to ATP content and survival curves were generated after normalizing for cell survival at 0 Gy for the respective controls. Values shown represent the mean ± SEM for three to four independent experiments. *P <* *0.05.

In the studies described here, GSCs were seeded at 250 or 500 cells/cm^2^ and radiosensitivity determined at 11 days after irradiation, 13 days after coculture initiation. At these initial cell numbers, GSCs seeded into the bottom of a trans-well chamber divide exponentially for at least 13 days with doubling times ranging from 36 to 48 h. Of note, the chambers were coated with po/ln, on which GSCs cultured alone (monoculture) or with astrocytes (coculture) grow as evenly dispersed monolayers, thus eliminating the complicating variable of different GSC growth patterns when cultured with and without astrocytes. Because the 11-day period corresponds to ∼4–7 cell doublings, all modes of cell death are reflected in the radiation survival assay. Important with respect to assay validity, inclusion of astrocytes on the top membrane did not affect GSC proliferation rate (Fig. S1A–D). As shown for GSCs grown as monocultures (Fig.1A–D), the dynamic range of the assay was greater than 2 logs of cell death. Comparison with the survival curves generated for GSCs grown in coculture indicates that their radiosensitivities were significantly reduced by the presence of astrocytes. The dose-modifying factors (DMFs) at a surviving fraction of 0.1 for NSC11, GBAM1, NSC23, and 923 were 1.37, 2.70, 1.49, and 1.37, respectively. Of note, the radiosensitivity of the long established glioma cell line U251 was not affected by coculture with astrocytes (Fig. S2), suggesting that the radioprotection provided by astrocytes may be selective for GSCs. These results are consistent with the microenvironment contributing to GBM radioresistance and suggest a specific role for astrocytes.

**Figure 2:**
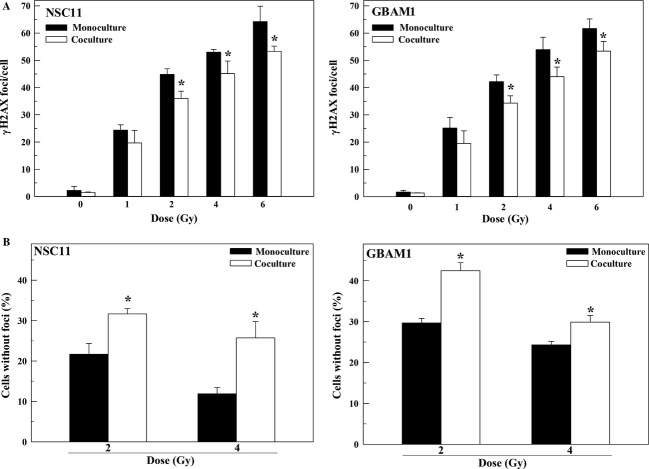
Influence of astrocytes on radiation-induced *γ*H2AX foci. Radiation was delivered 48 h after coculture initiation to glioblastoma stem-like cells alone (monoculture) or in coculture. (A) *γ*H2AX foci/cell at 1 h as a function radiation dose. (B) Percentage of cells with *γ*H2AX foci above unirradiated controls remaining at 24 h after irradiation. Values shown represent the mean ± SEM for three to four independent experiments. *P <* *0.05.

To begin to investigate the mechanisms responsible for the astrocyte-mediated radioprotection of GSCs, we focused on NSC11 and GBAM1. Astrocytes had no significant effect on the percentage of cells in S-phase in either GSC line after 48 h of coculture, the time of irradiation (Fig. S3), indicating that the reduction in GSC radiosensitivity induced by astrocytes cannot be attributed to redistribution into a radioresistant phase of the cell cycle. The critical lesion mediating radiation-induced cell death is the DNA DSB. Because *γ*H2AX nuclear foci correspond to radiation-induced DSBs and their dispersal correlates with DSB repair 12–14, *γ*H2AX foci levels were determined in the GSCs grown alone (monoculture) and in coculture with astrocytes (Fig.2). To determine the effects of astrocytes on the initial level of radiation-induced DSBs, *γ*H2AX foci were defined at 1 h as a function of radiation dose (Fig.2A). For both GSC lines the number of *γ*H2AX foci induced was greater in monoculture versus coculture with the difference reaching statistical significance at doses ≥2 Gy. We also defined the percentage of cells without *γ*H2AX foci at 24 h after irradiation, which reflects the number of cells that have completely repaired the induced DSBs. As shown in Figure2B, the percentage of cells without *γ*H2AX foci at 24 h was significantly greater in GSCs irradiated in coculture as compared to monocultures. These data suggest that the presence of astrocytes reduces the initial level of radiation-induced DSBs in GSCs and may enhance the repair of those that are induced, processes consistent with reduced radiosensitivity.

**Figure 3:**
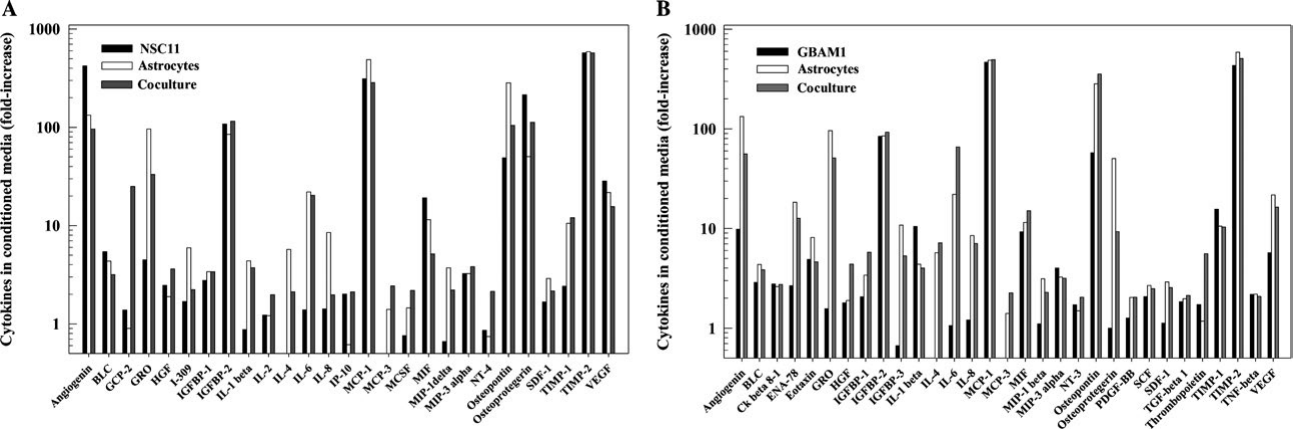
Cytokine profiles from glioblastoma stem-like cell, astrocytes, and coculture conditioned media as compared to stem cell medium: (A) NSC11, (B) GBAM1. Data generated from astrocytes alone are the same in (A) and (B). Of the 80 chemokines/cytokines evaluated, only those that were increased ≥twofold in cocultures are shown. Values shown represent the mean of two biological replicates.

The lack of direct contact between phenotypes in this coculture model indicates that the astrocyte-mediated radioprotection of GSCs involves a paracrine-based mechanism. Accordingly, chemokine/cytokine profiles were generated from media conditioned for 48 h on GSCs (NSC11 or GBAM1), astrocytes, and their respective cocultures. The cytokines/chemokines that were increased ≥twofold under coculture conditions as compared to stem cell media alone are shown in Figure3A and B. For NSC11 and GBAM1, of the 80 cytokines/chemokines evaluated 27 and 31, respectively, were increased in cocultures compared to stem cell media. While NSC11 and GBAM1 cells alone secrete a number of cytokines, it would be the bioactive molecules increased in astrocyte and coculture media that may contribute to the reduced radiosensitivity of the GSCs. For NSC11 coculture these cytokines include GCP-2, GRO, IL-1beta, IL-4, IL-6, MCP3, MCSF, MIP1-delta, NT-4, and TIMP-1. For GBAM1 the cytokines elevated included a number in common with the NSC11 cocultures (GRO, IL-4, IL-6, and MCP3) as well as a number unique to GBAM1 (ENA-78, IGFBP3, and osteoprotegerin). The complete list of the chemokines/cytokines for each cell line and condition is presented in Table S1. These data indicate that astrocyte cocultures provide a number of bioactive molecules that, acting individually or in combination, could influence the radiosensitivity of the GSCs.

**Figure 4:**
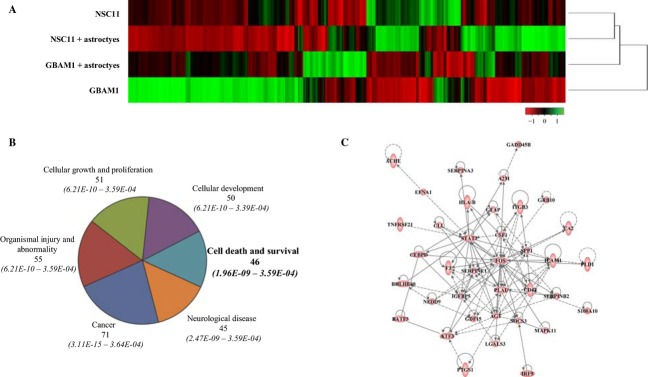
The effect of astrocyte coculture on GSC gene expression. (A) Unsupervised hierarchical clustered heatmap of row-normalized *z*-score values comparing probesets of NSC11 and GBAM1 monocultures versus GSCs exposed to astrocytes for 48 h (*P* < 0.05). (B) Commonly increased genes from NSC11 and GBAM1 cocultures (*P* < 0.05 and an increase of ≥twofold) were subjected to IPA analysis with the top six biological functions (containing 45 or more genes) shown. (C) IPA derived interconnecting network formed by 37 of the 46 genes from the cell death and survival category. GSCs, glioblastoma stem-like cells; IPA, ingenuity pathway analysis.

To further define the consequences of astrocytes on GSCs, gene expression profiles were generated from NSC11 and GBAM1 cells grown alone (monoculture) and compared to those generated 48 h after the initiation of coculture with astrocytes. As shown in the unsupervised hierarchical analysis of *z*-scores (Fig.4A), NSC11 and GBAM1 cells have disparate gene expression profiles that in general become more similar when grown in coculture with astrocytes. Although astrocytes induced changes in transcript levels specific to the individual GSC lines, there were a significant number of transcripts (1561) whose expression was commonly altered in NSC11 and GBAM1 cells as a result of coculture. To select transcripts for further analysis, an additional expression cut-off of twofold was applied. Of the 1561 transcripts whose expression was significantly modified in both the GSCs as a result of coculture with astrocytes (*P* < 0.05), 112 transcripts were increased at least twofold and 90 transcripts were downregulated to at least 0.5-fold (Table S2). These transcripts were subjected to IPA, which distributes genes into networks defined by known interactions and then matches these networks with specific biologically significant pathways. For the GSC transcripts increased after coculture with astrocytes, six biological functions (containing at least 45 molecules) were identified (Fig.4B). Because it contained signaling molecules previously associated with radioresponse, the *Cell Death and Survival* biological function was evaluated in more detail; 37 of the 46 transcripts distributed to an interconnecting network that included STAT3 and SOCS3, components of Jak/STAT signaling pathway, as well as FOS and MAPK11, components of the MAPK/JNK signaling pathway (Fig.4C). IPA was also used to identify *Upstream Regulators* of the 112 GSC transcripts that were increased in coculture (Table S3). The top regulator molecule was IL-6, which targets 26 of the 112 transcripts. The top 10 *Upstream Regulators* included additional cytokines and growth factors, consistent with a paracrine-based effect of astrocytes on GSC gene expression, as well as the transcription factors NFκB and STAT3, which are subject to regulation by cytokines/growth factors. IPA of the 90 transcripts that were decreased as a result of coculture produced only one biological function containing more than 45 molecules: *Cancer* (*P*-value 1.26E-07 to 4.85E-02).

**Figure 5:**
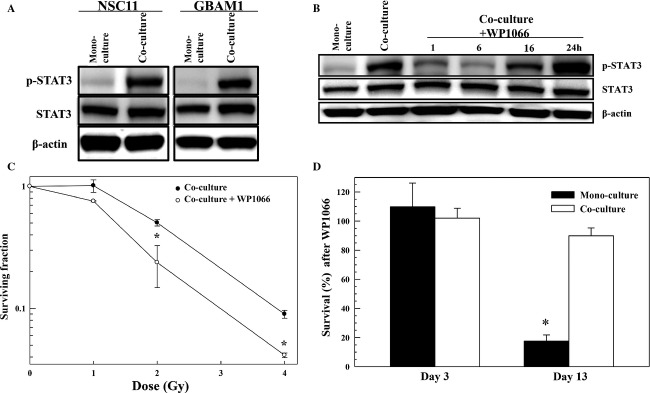
STAT3 as a target for glioblastoma stem-like cell radiosensitization in astrocyte coculture. (A) NSC11 and GBAM1 cells were grown alone or in coculture with astrocytes for 48 h and subjected to immunoblot analysis. Blots are representative of three to four independent experiments. (B) NSC11 cells grown for 48 h in coculture with astrocytes were treated with WP1060 (20 *μ*mol/L), collected at the specified time-points and subjected to immunoblot analysis. Blots are representative for two independent experiments. For the immunoblots shown *β*-actin was used as a loading control. (C) Influence of WP1060 on the radiosensitivity of NSC11 cells grown in astrocyte coculture. Forty-eight hours after coculture initiation WP1066 (20 *μ*mol/L) was added, 6 h later cultures were irradiated; fresh, drug-free media was added after 24 h and survival determined 11 days later. Values shown represent the mean ± SEM for three independent experiments. *P <* *0.05. (D) Effects of WP1066 alone on the survival of NSC11 cells grown in monoculture or in astrocyte coculture. Forty-eight hours after coculture or monoculture initiation, WP1066 (20 *μ*mol/L) was added to both growth conditions; 30 h later cells were collected for survival analysis or fresh, drug-free media was added and survival determined 11 days later. Values shown represent the mean ± SEM for three independent experiments. *P <* *0.05.

These results suggest that astrocytes modify GSC gene and chemokine expression and thus phenotype. Such changes in chemokine and gene expression may provide insights into the mechanisms mediating the radioprotective effect of astrocytes on GSCs and, moreover, suggest additional targets for radiosensitization, that is, targets that would not be identified based solely on studies of GSCs in monoculture. As proof of principle, we initially addressed the role of STAT3 in GSC radiosensitivity. This transcription factor is regulated by IL-6, among a number of other cytokines 26 whose presence is increased in coculture conditioned media its mRNA levels were increased as a result of coculture with astrocytes (Fig.4C) and based on IPA it is predicted to be activated in coculture (Table S3). Immunoblot analyses of STAT3 and p-STAT3 in GSCs alone and in their respective astrocyte cocultures are shown in Figure5. Total STAT3 levels were only slightly increased in NSC11 and GBAM1 in coculture as compared to monoculture (1.18 ± 0.01 and 1.5 ± 0.4, respectively, *n* = 3–4). However, the level of p-STAT3 (activated) was clearly elevated in the GSCs cocultured with astrocytes (Fig.5A) consistent with the IPA results.

To test the hypothesis that STAT3 contributes to the astrocyte-mediated decrease in GSC radiosensitivity, we used WP1066, which inhibits STAT3 activation 28. Addition of WP1066 (20 *μ*mol/L) to NSC11/astrocyte cocultures reduced p-STAT3 levels by 1 h reaching a maximum decrease by ∼6 h and returning to untreated levels by 24 h (Fig.5B). To determine the effects of WP1066 on the radiosensitivity of NSC11when cultured with astrocytes, 2 days after coculture initiation, drug (20 *μ*mol/L) was added 6 h before irradiation with fresh, drug-free media added after 24 h; survival was then determined 11 days later. As shown in Figure5C, addition of WP1066 enhanced NSC11 radiosensitivity (DEF of 1.4). Attempts to use this same protocol on NSC11 in monoculture were complicated by the excessive toxicity induced by WP1066 treatment alone (Fig.5D). Whereas no reduction in NSC11 survival was detected immediately after the 30 h WP1066 exposure period in mono- or cocultures, 11 days after treatment survival of NSC11 cells in monoculture was reduced by greater than 80% as compared to no significant reduction in survival of NSC11 treated in coculture. These data suggest that WP1066 is an effective cytotoxic agent against NSC11 cells grown alone, yet it has little effect on these GSCs when grown in astrocyte coculture.

**Figure 6:**
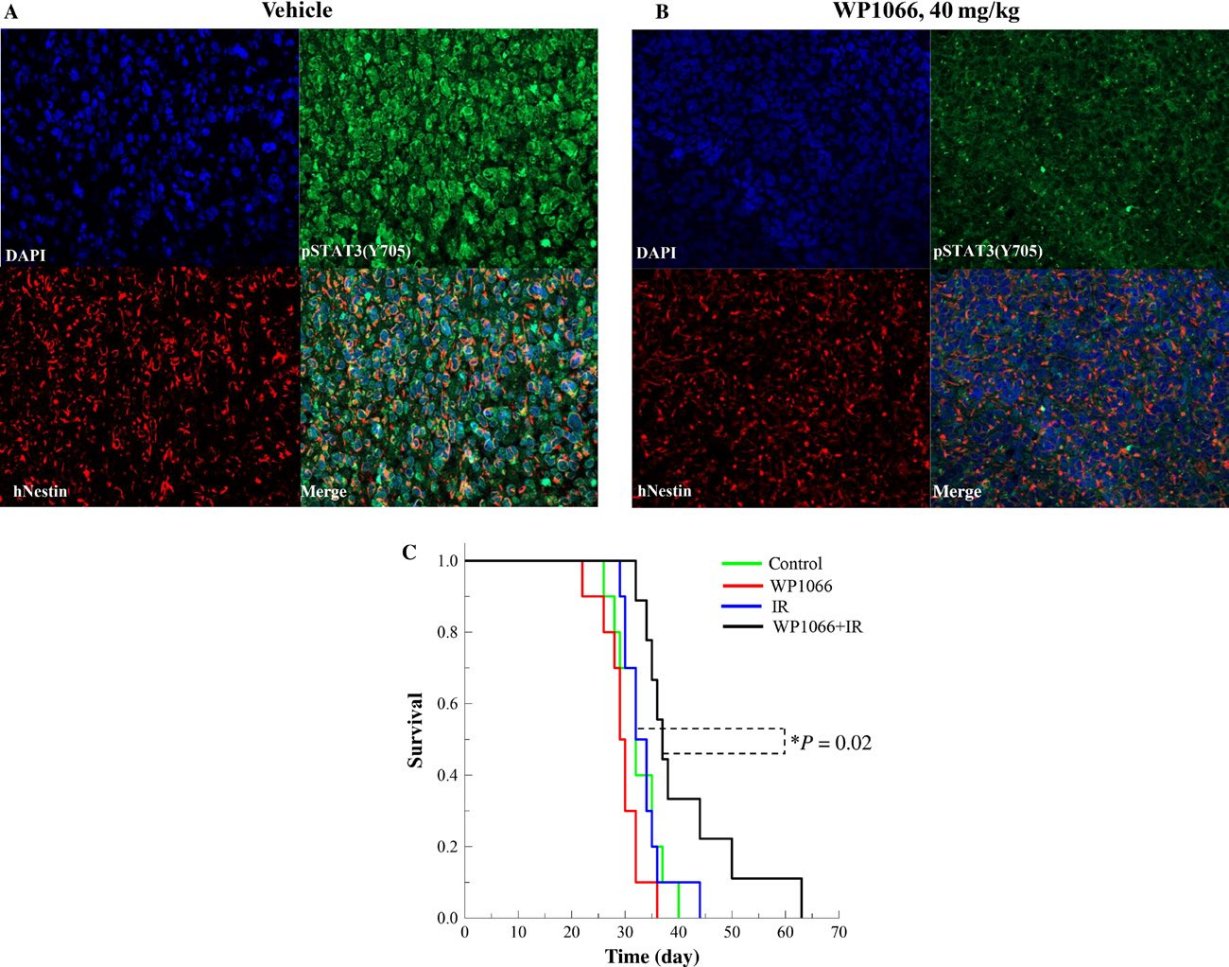
Influence of WP1066 on the radioresponse of orthotopic xenografts initiated from CD133+ NSC11 cells. At 24 days after orthotopic implant, mice were randomized and treatment initiated as described. Mice were followed until the onset of morbidity. At the onset of morbidity (mean 57 days), mice bearing orthotopic xenografts were exposed to vehicle (A) or WP1066 (40 mg/kg), oral gavage (B) and collected 6 h later for immunohistochemical evaluation of p-STAT3 (Y705; green), Nestin to identify human tumor cells (red), and nuclei (blue), 20× magnification. (C) Kaplan–Meier survival curves were generated with log-rank analysis for comparison.

To determine whether the WP1066-induced radiosensitization of GSCs grown in coculture with astrocytes translated to an orthotopic model, NSC11 cells were used to initiate ic xenografts 10. Initially, the ability of WP1066 to decrease phosphorylation of STAT3 in NSC11 orthotopic xenograft was tested. At the onset of tumor-induced morbidity, WP1066 (40 mg/kg) was delivered by oral gavage; brains were collected 6 h later and subjected to immunofluoresent histochemical analysis. Sections were obtained from non-necrotic portions of the tumor. As shown in Figure6A, p-STAT3 was clearly detectable in brain tumor xenografts from vehicle-treated mice, whereas WP1066 treatment clearly reduced the level of p-STAT3, indicative of the inhibition of STAT3 activity. Furthermore, treatment of mice with WP1066 had no effect on the expression of total STAT3 (Fig. S4). Because of its ability to reduce p-STAT3 levels in the NSC11 orthotopic xenografts, the effect of WP1066 on the radioresponse of these brain tumors was determined. Specifically, mice bearing NSC11 orthotopic tumors were randomized according to BLI signal into four groups: vehicle (control), radiation (12 Gy), WP1066 (40 mg/kg) 23, and WP1066 plus radiation. WP1066 was delivered once a day (40 mg/kg, oral gavage) for 3 days with the tumor locally irradiated (12 Gy) 6 h after each drug treatment. Mice were followed until the initial onset of morbidity and Kaplan–Meier survival curve was generated (Fig.6C). While WP1066 or radiation alone had no significant effect on mouse survival as compared to control treatment (*P* = 0.08 and 0.80, respectively), the survival of mice receiving the combination protocol (WP1066 plus radiation) was significantly increased as compared to control (*P* = 0.02) and importantly, as compared to radiation alone (*P* = 0.02). Thus, these data indicate that WP1066 enhances the radioresponse of NSC11 orthotopic xenografts, as predicted by the in vitro coculture model.

## Discussion

Attempts to define the mechanisms responsible for GBM radioresistance have focused almost entirely on the use of in vitro tumor cell cultures. However, the relatively homogeneous radioresistance of GBMs in a background of intertumor heterogeneity suggests that the microenvironment may play a significant role in determining their radioresponse. Moreover, recent results comparing the radioresponse of GSCs grown in vitro and as orthotopic xenografts also suggest a microenvironmental contribution to GBM radioresistance 10,11. Toward developing an expedient in vitro model for investigating GBM radiosensitivity under conditions that begin to account for the microenvironment, the studies described here used the coculture of GSCs with normal human astrocytes. Astrocytes are known to regulate the activities of other normal central nervous system (CNS) components modulating brain function and response to injury 15–18. With respect to tumor cells, coculture of astrocytes with breast and lung tumor cell lines 28 as well as melanoma cells 29 reduced their sensitivity to a variety of chemotherapeutic agents, which was attributed to a decrease in apoptosis. Moreover, astrocytes have been reported to protect a lymphoma cell line from radiation-induced apoptosis 30. As shown here, astrocytes reduced the in vitro radiosensitivity of a panel human GSCs. However, because it is an infrequent mode of cell death in irradiated GSCs 9,31, consistent with GBM and most solid tumor cells 32, in contrast to previous reports, a reduction in apoptosis is unlikely to contribute to the astrocyte-mediated GSC radioprotection.

The mechanism through which astrocytes decrease GSC radiosensitivity appears to involve the modulation of radiation-induced DSBs. The presence of astrocytes reduced the initial level of radiation-induced *γ*H2AX expression, indicative of a reduction in radiation-induced DSBs 12–14. In addition, fewer GSCs in coculture continued to express *γ*H2AX at 24 h after irradiation as compared to GSCs grown alone, which suggests that the presence of astrocytes also enhanced the repair of DSBs 13,14. While the specific molecular processes responsible remain to be defined, the reduced *γ*H2AX foci induction and their enhanced dispersal are consistent with a decrease in radiosensitivity. Of note, these effects are similar, although not to the same degree, as found for GSCs irradiated in vitro as compared to those irradiated as orthotopic xenografts 10,11.

The conditions of the coculture assay indicate that the astrocyte-mediated decrease in GSC radiosensitivity was the result of a paracrine-based process. Along these lines, a number of cytokines already associated with radioresistance such as HGF 33, IL-6 34, IL-8 35, and TGF*β* 36 as well as others yet to be investigated in terms of modifying radioresponse were found to be increased in the GSC/astrocyte cocultures as compared to GSCs alone. Given the number of chemokines/cytokines increased in cocultures, it would seem that multiple paracrine factors acting individually, in combination, and/or in a redundant manner could contribute to the regulation of GSC radiosensitivity. Exposure to the astrocyte-generated cytokines/chemokines resulted in significant changes in GSC gene expression profiles consistent with coculture-induced phenotypic modifications. Moreover, while there were GSCs line-specific changes in gene expression (Fig.3A), genes whose expression were increased in both NSC11 and GBAM1 cells as a result of astrocyte coculture included those associated with the JAK/STAT, MAPK/JNK, and NFκB signaling networks, which have each been linked to radioresistance 37–40. These data suggest that cytokines provided by the microenvironment and the accompanying changes in tumor cell gene expression may play a role in determining GBM radioresponse.

In addition to fundamental investigations into the processes through which the microenvironment can mediate radioresistance, a goal of these studies was to determine whether this coculture system could provide unique insights into the targets for GBM radiosensitization. Toward this end, cytokine and gene expression analyses suggested that the activation of STAT3 was substantially increased in NSC11 and GBAM1 cells when cocultured with astrocytes, which was validated by immunoblot analysis. Under standard monoculture conditions, pSTAT3 was expressed at very low levels and importantly, treatment of NSC11 cells with the JAK/STAT3 inhibitor WP1066 alone resulted in a significant level of cytotoxicity. Thus, in search of targets for GBM radiosensitization, these two data sets would not have supported further evaluation of STAT3 activation. In contrast, under coculture conditions p-STAT3 was clearly detectable in GSCs and treatment with WP1066 alone had no cytotoxic effect. These results combined with previous reports indicating that the inhibition of STAT3 activation can enhance the radiosensitivity of certain tumor cell lines 41,42 provided the rationale for testing the effects of WP1066 on NSC11 cells grown in coculture with astrocytes and in vivo as orthotopic xenografts. Under both experimental conditions, while WP1066 alone had no detectable cytotoxic effect on NSC11 cells, the JAK/STAT3 inhibitor did enhance their radiosensitivity. Given that WP1066 has recently entered clinical trials as a single modality treatment for brain cancer (http://clinicaltrials.gov/show/NCT01904123), these data support the study of its combination with radiotherapy against GBM. Moreover, these results illustrate that simulating the GBM microenvironment in vitro has the potential to identify novel targets for radiosensitization (i.e., those not suggested from standard culture conditions) applicable to an in situ setting. Whether GSCs in coculture with astrocytes are more resistant to other DNA-damaging and molecularly targeted agents remains to be determined.

## Conflict of Interest

None declared.

## Supporting Information

**Figure S1.** Effect of astrocytes on GSC proliferation: A) NSC11, B) GBAM1, C) NSC23, D) 923. GSCs were seeded at densities of 500cells/cm2 (NSC11, GBAM1) or 250cells/cm2 (NSC23, 923) onto po/ln coated 24well-plates and astrocytes (1.33x106cells/cm2) in trans-wells suited for 24well-plates, respectively and allow to attach over night. Cell proliferation was defined at indicated time-points according to ATP content (CellTiter-Glow, Promega) and proliferation curves generated. Values shown represent the mean ± STDEV for 3 independent experiments. P-value was determined by Mann-Whitney rank sum test.

**Figure S2.** Effect of astrocytes on U251 radiosensitivity: 48h after co-culture initiation mono- and co-cultures were irradiated and 11 days later cell number was determined according to ATP content and survival curves were generated after normalizing for cell survival at 0Gy for the respective controls. Values shown represent the means ± SEM for 4 independent experiments.

**Figure S3.** Effect of astrocytes on GSC cell cycle distribution: 48h after co-culture initiation mono-and co-cultures of NSC11 and GBAM1 were collected for fixation, stained with propidium iodide, and analyzed by flow cytometry with cell cycle distributions determined using FlowJo Software (Tree Star, Inc.). All cultures were sub-confluent at the time of collection. Values shown represent the means for 3 independent experiments.

**Figure S4.** Influence of WP1066 on the expression of total-STAT3 in orthotopic xenografts. A) and B) At the onset of morbidity (mean 57 days), mice bearing orthotopic xenografts were exposed to vehicle (A) or WP1066 (40mg/kg, oral gavage; B) and collected 6 hours later for immunohistochemical evaluation of total-STAT3 (green), Nestin to identify human tumor cells (red), and nuclei (blue), 20× magnification.

**Table S1.** Chemokine/cytokine profile from NSC11, GBAM1, astrocytes, and cocultures of NSC11 and GBAM1. Values shown represent the level of proteins in conditioned media compared to stem cell media alone.

**Table S2.** Commonly affected genes (202) in NSC11 and GBAM1 GSCs after indirect coculture with astrocytes, increased or decreased at least twofold. Log twofold changes are displayed.

**Table S3.** Top 10 upstream regulators of the 112 transcripts whose expression was increased (≥twofold, *P* ≤ 0.05) in cocultured GSCs compared to monocultures.
