# Supplementary material for: Coculture with astrocytes reduces the radiosensitivity of glioblastoma stem-like cells and identifies additional targets for radiosensitization
Source: Cancer Med. 2015 Oct 30;4(11):1705–16. doi: 10.1002/cam4.510 (PMC4673998; doi:10.1002/cam4.510)
Supplement: Supplementary file 5 [file cam40004-1705-sd5.docx]

**Table S1:** Chemokine/Cytokine profile from NSC11, GBAM1, astrocytes and co-cultures of NSc11 and GBAM1. Values shown represent the level of proteins in conditioned media compared to stem cell media alone.

| **Name** | **NSC11**  **(mono-culture)** | **GBAM1**  **(mono-culture)** | **astrocytes**  **(mono-culture)** | **NSC11**  **(co-culture)** | **GBAM1**  **(co-culture)** |
| --- | --- | --- | --- | --- | --- |
| **Angiogenin** | 421.0 | 9.8 | 133.4 | 95.9 | 55.9 |
| **BDNF** | 1.8 | 1.1 | 1.3 | 1.1 | 0.8 |
| **BLC** | 5.4 | 2.9 | 4.3 | 3.2 | 3.9 |
| **Ck beta 8-1** | 2.6 | 2.8 | 2.6 | 1.8 | 2.8 |
| **EGF** | 0.8 | 0.8 | 0.8 | 0.7 | 0.8 |
| **ENA-78** | 0.9 | 1.7 | 10.4 | 1.8 | 7.1 |
| **Eotaxin** | 2.8 | 2.6 | 4.8 | 1.4 | 2.8 |
| **Eotaxin-2** | 0.9 | 0.6 | 0.9 | 0.9 | 1.0 |
| **Eotaxin-3** | 0.1 | 0.1 | 0.1 | 1.2 | 0.1 |
| **FGF-4** | 0.7 | 0.1 | 0.8 | 1.0 | 0.6 |
| **FGF-6** | 1.4 | 1.3 | 2.5 | 1.5 | 1.6 |
| **FGF-7** | 0.2 | 0.3 | 1.0 | 0.8 | 0.4 |
| **FGF-9** | 0.3 | 0.3 | 1.5 | 0.8 | 0.6 |
| **Flt-3 Ligand** | 1.4 | 1.0 | 1.2 | 1.5 | 1.4 |
| **Fractalkine** | 1.9 | 0.2 | 0.6 | 1.2 | 0.1 |
| **GCP-2** | 1.4 | 0.9 | 0.9 | 25.0 | 0.3 |
| **G-CSF** | 1.9 | 1.8 | 2.1 | 1.4 | 1.9 |
| **GDNF** | 1.9 | 1.3 | 0.9 | 1.1 | 1.1 |
| **GM-CSF** | 1.3 | 1.3 | 1.7 | 1.3 | 1.7 |
| **GRO** | 4.5 | 1.6 | 96.0 | 33.2 | 50.9 |
| **GRO-alpha** | 1.5 | 1.5 | 1.6 | 1.1 | 1.7 |
| **HGF** | 2.5 | 1.8 | 1.9 | 3.6 | 4.4 |
| **I-309** | 1.7 | 1.0 | 5.9 | 2.2 | 1.1 |
| **IFN-gamma** | 1.2 | 1.2 | 1.2 | 1.1 | 1.3 |
| **IGFBP-1** | 2.8 | 2.1 | 3.4 | 3.4 | 5.8 |
| **IGFBP-2** | 108.0 | 84.2 | 84.9 | 115.7 | 92.8 |
| **IGFBP-3** | 5.0 | 0.7 | 10.8 | 1.7 | 5.3 |
| **IGFBP-4** | 0.7 | 0.4 | 0.7 | 1.7 | 1.0 |
| **IGF-I** | 1.8 | 1.6 | 1.5 | 1.0 | 1.8 |
| **IL-1 alpha** | 1.3 | 0.9 | 1.2 | 1.1 | 1.2 |
| **IL-1 beta** | 0.9 | 10.5 | 4.4 | 3.7 | 4.0 |
| **IL-10** | 1.4 | 1.3 | 1.6 | 1.0 | 1.4 |
| **IL-12 p70** | 0.8 | 1.4 | 1.1 | 0.9 | 1.0 |
| **IL-13** | 0.7 | 0.9 | 0.7 | 0.8 | 1.0 |
| **IL-15** | 1.8 | 1.4 | 1.2 | 1.4 | 1.5 |
| **IL-16** | 0.6 | 0.8 | 1.1 | 1.0 | 0.8 |
| **IL-2** | 1.2 | 1.1 | 1.2 | 2.0 | 1.3 |
| **IL-3** | 1.2 | 1.4 | 1.2 | 1.4 | 1.7 |
| **IL-4** | 0.5 | 0.5 | 5.7 | 2.1 | 7.2 |
| **IL-5** | 0.4 | 0.5 | 0.5 | 0.8 | 0.5 |
| **IL-6** | 1.4 | 1.1 | 22.0 | 20.3 | 65.7 |
| **IL-7** | 0.8 | 0.7 | 0.9 | 1.0 | 0.8 |
| **IL-8** | 1.4 | 1.2 | 8.5 | 2.0 | 7.1 |
| **IP-10** | 2.0 | 0.6 | 0.6 | 2.1 | 0.6 |
| **Leptin** | 0.5 | 0.5 | 0.3 | 0.8 | 0.4 |
| **LIF** | 0.8 | 0.9 | 0.9 | 0.8 | 0.7 |
| **LIGHT** | 1.1 | 0.4 | 1.3 | 0.7 | 0.9 |
| **MCP-1** | 311.5 | 467.2 | 489.5 | 285.5 | 495.6 |
| **MCP-2** | 0.8 | 0.3 | 0.7 | 1.3 | 0.2 |
| **MCP-3** | 0.1 | 0.3 | 1.4 | 2.4 | 2.3 |
| **MCP-4** | 0.5 | 0.3 | 0.5 | 1.4 | 1.2 |
| **MCSF** | 0.8 | 0.7 | 1.5 | 2.2 | 0.7 |
| **MDC** | 2.2 | 0.4 | 1.5 | 0.8 | 0.7 |
| **MIF** | 19.2 | 9.3 | 11.5 | 5.1 | 15.0 |
| **MIG** | 1.1 | 1.0 | 1.3 | 1.5 | 1.1 |
| **MIP-1 beta** | 1.2 | 1.1 | 3.1 | 0.5 | 2.3 |
| **MIP-1delta** | 0.7 | 1.1 | 3.7 | 2.2 | 1.7 |
| **MIP-3 alpha** | 3.3 | 4.0 | 3.3 | 3.8 | 3.2 |
| **NAP-2** | 1.4 | 1.1 | 0.7 | 1.4 | 1.4 |
| **NT-3** | 2.0 | 1.7 | 1.5 | 1.3 | 2.0 |
| **NT-4** | 0.9 | 1.2 | 0.7 | 2.1 | 1.2 |
| **Oncostatin M** | 1.0 | 0.6 | 1.0 | 0.7 | 0.7 |
| **Osteopontin** | 48.8 | 57.4 | 283.3 | 104.9 | 354.5 |
| **Osteoprotegerin** | 214.2 | 1.0 | 50.3 | 112.7 | 9.3 |
| **PARC** | 0.8 | 0.7 | 0.8 | 0.9 | 0.7 |
| **PDGF-BB** | 1.1 | 1.3 | 2.0 | 1.5 | 2.0 |
| **PIGF** | 1.0 | 1.6 | 1.0 | 1.0 | 1.6 |
| **RANTES** | 1.0 | 0.7 | 3.0 | 1.3 | 1.1 |
| **SCF** | 2.4 | 2.1 | 2.7 | 1.4 | 2.5 |
| **SDF-1** | 1.7 | 1.1 | 2.9 | 2.2 | 2.6 |
| **TARC** | 2.3 | 1.9 | 1.4 | 1.3 | 1.8 |
| **TGF-beta 1** | 1.9 | 1.8 | 2.0 | 1.4 | 2.1 |
| **TGF-beta 2** | 0.6 | 0.6 | 0.4 | 0.7 | 0.6 |
| **TGF-beta 3** | 1.5 | 1.9 | 1.3 | 1.1 | 1.5 |
| **Thrombopoietin** | 0.5 | 1.7 | 1.2 | 1.5 | 5.6 |
| **TIMP-1** | 2.4 | 15.6 | 10.6 | 12.1 | 10.3 |
| **TIMP-2** | 573.0 | 301.1 | 586.7 | 571.9 | 509.4 |
| **TNF-alpha** | 1.8 | 1.4 | 1.6 | 1.7 | 1.6 |
| **TNF-beta** | 2.5 | 2.2 | 2.2 | 1.3 | 2.1 |
| **VEGF** | 28.5 | 5.7 | 21.7 | 15.6 | 16.4 |
