# Supplementary material for: Coculture with astrocytes reduces the radiosensitivity of glioblastoma stem-like cells and identifies additional targets for radiosensitization
Source: Cancer Med. 2015 Oct 30;4(11):1705–16. doi: 10.1002/cam4.510 (PMC4673998; doi:10.1002/cam4.510)
Supplement: Supplementary file 6 [file cam40004-1705-sd6.docx]

**Supplemental Table 2:** Commonly affected genes (202) in NSC11 and GBAM1 GSCs after indirect co-culture with astrocytes, increased or decreased at least 2-fold. Log2-fold changes are displayed.

| **Probe Set ID** | **Entrez GeneID** | **Gene** | **Description** | **log2 fold-change GBAM1 co-culture vs. GBAM1 mono-culture** | **log2 fold-change NSC11 co-culture vs. NSC11 mono-culture** |
| --- | --- | --- | --- | --- | --- |
| 214110_s_at | 0 | 0 | CDNA clone IMAGE:4801360 | 2.6 | 2.0 |
| 212847_at | 0 | 0 | MRNA; cDNA DKFZp779L1713 (from clone DKFZp779L1713) | -1.2 | -1.1 |
| 214814_at | 0 | 0 | Transcribed locus, weakly similar to NP_596914.1 YTH domain-containing protein 1 [Rattus norvegicus] | -1.7 | -1.2 |
| 215993_at | 0 | 0 | Clone 24740 mRNA sequence | -1.8 | -3.3 |
| 215763_at | 0 | 0 | Transcribed locus | -1.9 | -3.2 |
| 215164_at | 0 | 0 | Transcribed locus | -2.2 | -2.2 |
| 216822_x_at | 0 | 0 | 0 | -2.4 | -2.0 |
| 217757_at | 2 | A2M | alpha-2-macroglobulin (A2M), mRNA. | 7.3 | 23.1 |
| 214829_at | 10157 | AASS | aminoadipate-semialdehyde synthase (AASS), nuclear gene encoding mitochondrial protein, mRNA. | -1.8 | -1.2 |
| 205377_s_at | 43 | ACHE | acetylcholinesterase (ACHE), transcript variant E4-E6, mRNA. | 2.2 | 4.2 |
| 205364_at | 8309 | ACOX2 | acyl-CoA oxidase 2, branched chain (ACOX2), mRNA. | 3.7 | 2.0 |
| 202834_at | 183 | AGT | angiotensinogen (serpin peptidase inhibitor, clade A, member 8) (AGT), mRNA. | 6.1 | 2.9 |
| 202588_at | 203 | AK1 | adenylate kinase 1 (AK1), mRNA. | 2.3 | 2.5 |
| 213004_at | 23452 | ANGPTL2 | angiopoietin-like 2 (ANGPTL2), mRNA. | 2.1 | 2.4 |
| 212286_at | 23253 | ANKRD12 | ankyrin repeat domain 12 (ANKRD12), transcript variant 2, mRNA. | -1.8 | -1.1 |
| 203525_s_at | 324 | APC | adenomatous polyposis coli (APC), transcript variant 3, mRNA. | -1.6 | -1.1 |
| 213138_at | 10865 | ARID5A | AT rich interactive domain 5A (MRF1-like) (ARID5A), mRNA. | 2.5 | 2.2 |
| 202206_at | 10123 | ARL4C | ADP-ribosylation factor-like 4C (ARL4C), mRNA. | 2.8 | 3.5 |
| 202207_at | 10123 | ARL4C | ADP-ribosylation factor-like 4C (ARL4C), mRNA. | 2.8 | 5.5 |
| 202208_s_at | 10123 | ARL4C | ADP-ribosylation factor-like 4C (ARL4C), mRNA. | 2.3 | 5.2 |
| 219973_at | 79642 | ARSJ | arylsulfatase family, member J (ARSJ), mRNA. | 2.4 | 2.8 |
| 219918_s_at | 259266 | ASPM | asp (abnormal spindle) homolog, microcephaly associated (Drosophila) (ASPM), transcript variant 2, mRNA. | -2.2 | -1.0 |
| 202672_s_at | 467 | ATF3 | Activating transcription factor 3 | 3.1 | 5.9 |
| 220237_at | 64422 | ATG3 | autophagy related 3 (ATG3), mRNA. | -1.1 | -1.3 |
| 210121_at | 8707 | B3GALT2 | UDP-Gal:betaGlcNAc beta 1,3-galactosyltransferase, polypeptide 2 (B3GALT2), mRNA. | -1.8 | -2.7 |
| 220358_at | 55509 | BATF3 | basic leucine zipper transcription factor, ATF-like 3 (BATF3), mRNA. | 3.7 | 3.0 |
| 205363_at | 8424 | BBOX1 | butyrobetaine (gamma), 2-oxoglutarate dioxygenase (gamma-butyrobetaine hydroxylase) 1 (BBOX1), mRNA. | 3.9 | 3.1 |
| 214499_s_at | 9774 | BCLAF1 | BCL2-associated transcription factor 1 (BCLAF1), transcript variant 2, mRNA. | -1.1 | -1.4 |
| 201101_s_at | 9774 | BCLAF1 | BCL2-associated transcription factor 1 (BCLAF1), transcript variant 3, mRNA. | -1.1 | -1.0 |
| 201170_s_at | 8553 | BHLHE40 | basic helix-loop-helix family, member e40 (BHLHE40), mRNA. | 6.4 | 4.0 |
| 202201_at | 645 | BLVRB | biliverdin reductase B (flavin reductase (NADPH)) (BLVRB), mRNA. | 6.5 | 3.1 |
| 203840_at | 8548 | BLZF1 | Basic leucine zipper nuclear factor 1 | -1.4 | -2.1 |
| 214727_at | 675 | BRCA2 | breast cancer 2, early onset (BRCA2), mRNA. | -1.4 | -1.4 |
| 221703_at | 83990 | BRIP1 | BRCA1 interacting protein C-terminal helicase 1 (BRIP1), mRNA. | -1.5 | -1.2 |
| 209183_s_at | 11067 | C10orf10 | chromosome 10 open reading frame 10 (C10orf10), mRNA. | 2.1 | 34.0 |
| 218614_at | 55196 | C12orf35 | chromosome 12 open reading frame 35 (C12orf35), mRNA. | -1.7 | -1.0 |
| 212067_s_at | 715 | C1R | complement component 1, r subcomponent (C1R), mRNA. | 2.2 | 2.4 |
| 218983_at | 51279 | C1RL | complement component 1, r subcomponent-like (C1RL), mRNA. | 2.3 | 2.6 |
| 209301_at | 760 | CA2 | carbonic anhydrase II (CA2), mRNA. | 2.2 | 3.5 |
| 205626_s_at | 793 | CALB1 | calbindin 1, 28kDa (CALB1), mRNA. | -1.6 | -3.2 |
| 220175_s_at | 644019 | CBWD6 | COBW domain containing 6 (CBWD6), mRNA. | -1.1 | -1.0 |
| 205034_at | 9134 | CCNE2 | cyclin E2 (CCNE2), mRNA. | -1.1 | -1.2 |
| 211814_s_at | 9134 | CCNE2 | cyclin E2 (CCNE2), mRNA. | -1.2 | -1.0 |
| 204489_s_at | 960 | CD44 | CD44 molecule (Indian blood group) | 5.1 | 4.8 |
| 214803_at | 1004 | CDH6 | cadherin 6, type 2, K-cadherin (fetal kidney) (CDH6), mRNA. | -1.6 | -1.1 |
| 203973_s_at | 1052 | CEBPD | CCAAT/enhancer binding protein (C/EBP), delta (CEBPD), mRNA. | 3.4 | 12.0 |
| 203341_at | 10153 | CEBPZ | CCAAT/enhancer binding protein (C/EBP), zeta (CEBPZ), mRNA. | -1.4 | -1.1 |
| 205046_at | 1062 | CENPE | centromere protein E, 312kDa (CENPE), mRNA. | -2.8 | -1.2 |
| 209172_s_at | 1063 | CENPF | centromere protein F, 350/400kDa (mitosin) (CENPF), mRNA. | -1.7 | -1.2 |
| 218566_s_at | 26973 | CHORDC1 | cysteine and histidine-rich domain (CHORD) containing 1 (CHORDC1), transcript variant 1, mRNA. | -1.2 | -1.3 |
| 203921_at | 9435 | CHST2 | carbohydrate (N-acetylglucosamine-6-O) sulfotransferase 2 (CHST2), mRNA. | 4.6 | 2.2 |
| 221065_s_at | 64377 | CHST8 | carbohydrate (N-acetylgalactosamine 4-0) sulfotransferase 8 (CHST8), transcript variant 1, mRNA. | 2.7 | 2.9 |
| 208791_at | 1191 | CLU | clusterin (CLU), transcript variant 1, mRNA. | 2.3 | 2.0 |
| 203951_at | 1264 | CNN1 | Calponin 1, basic, smooth muscle | 8.3 | 2.3 |
| 209716_at | 1435 | CSF1 | colony stimulating factor 1 (macrophage) (CSF1), transcript variant 3, mRNA. | 2.2 | 2.6 |
| 220462_at | 80034 | CSRNP3 | cysteine-serine-rich nuclear protein 3 (CSRNP3), transcript variant 2, mRNA. | -1.3 | -1.1 |
| 207244_x_at | 1548 | CYP2A6 | cytochrome P450, family 2, subfamily A, polypeptide 6 (CYP2A6), mRNA. | -1.3 | -1.1 |
| 203791_at | 1657 | DMXL1 | Dmx-like 1 (DMXL1), mRNA. | -1.6 | -1.1 |
| 202023_at | 1942 | EFNA1 | ephrin-A1 (EFNA1), transcript variant 2, mRNA. | 2.9 | 10.2 |
| 203729_at | 2014 | EMP3 | epithelial membrane protein 3 (EMP3), mRNA. | 2.3 | 3.0 |
| 218859_s_at | 51575 | ESF1 | ESF1, nucleolar pre-rRNA processing protein, homolog (S. cerevisiae) (ESF1), mRNA. | -1.9 | -1.2 |
| 219216_at | 54465 | ETAA1 | Ewing tumor-associated antigen 1 (ETAA1), mRNA. | -1.3 | -1.1 |
| 204363_at | 2152 | F3 | coagulation factor III (thromboplastin, tissue factor) (F3), transcript variant 2, mRNA. | 3.5 | 2.9 |
| 219694_at | 54491 | FAM105A | family with sequence similarity 105, member A (FAM105A), mRNA. | -1.0 | -1.2 |
| 216682_s_at | 55578 | FAM48A | Family with sequence similarity 48, member A | -1.4 | -1.3 |
| 206857_s_at | 2281 | FKBP1B | FK506 binding protein 1B, 12.6 kDa (FKBP1B), transcript variant 1, mRNA. | 2.2 | 2.1 |
| 209189_at | 2353 | FOS | FBJ murine osteosarcoma viral oncogene homolog (FOS), mRNA. | 3.4 | 7.8 |
| 218084_x_at | 53827 | FXYD5 | FXYD domain containing ion transport regulator 5 (FXYD5), transcript variant 2, mRNA. | 5.4 | 13.2 |
| 220131_at | 53822 | FXYD7 | FXYD domain containing ion transport regulator 7 (FXYD7), mRNA. | 5.4 | 7.0 |
| 206136_at | 7855 | FZD5 | frizzled family receptor 5 (FZD5), mRNA. | 4.9 | 4.8 |
| 221245_s_at | 7855 | FZD5 | frizzled family receptor 5 (FZD5), mRNA. | 3.2 | 2.8 |
| 209305_s_at | 4616 | GADD45B | growth arrest and DNA-damage-inducible, beta (GADD45B), mRNA. | 2.5 | 3.4 |
| 207574_s_at | 4616 | GADD45B | growth arrest and DNA-damage-inducible, beta (GADD45B), mRNA. | 2.3 | 3.3 |
| 204374_s_at | 2584 | GALK1 | galactokinase 1 (GALK1), mRNA. | 6.3 | 2.0 |
| 204471_at | 2596 | GAP43 | growth associated protein 43 (GAP43), transcript variant 1, mRNA. | 2.5 | 6.5 |
| 221577_x_at | 9518 | GDF15 | growth differentiation factor 15 (GDF15), mRNA. | 5.6 | 2.3 |
| 203540_at | 2670 | GFAP | glial fibrillary acidic protein (GFAP), transcript variant 1, mRNA. | 12.6 | 16.5 |
| 210999_s_at | 2887 | GRB10 | growth factor receptor-bound protein 10 (GRB10), transcript variant 1, mRNA. | 2.1 | 3.0 |
| 205523_at | 1404 | HAPLN1 | hyaluronan and proteoglycan link protein 1 (HAPLN1), mRNA. | -1.6 | -3.5 |
| 209140_x_at | 3106 | HLA-B | major histocompatibility complex, class I, B (HLA-B), mRNA. | 2.2 | 2.3 |
| 216526_x_at | 3107 | HLA-C | major histocompatibility complex, class I, C (HLA-C), transcript variant 2, mRNA. | 2.3 | 2.7 |
| 208812_x_at | 3107 | HLA-C | major histocompatibility complex, class I, C (HLA-C), transcript variant 1, mRNA. | 2.2 | 2.5 |
| 214459_x_at | 3107 | HLA-C | Major histocompatibility complex, class I, C | 2.1 | 2.6 |
| 204754_at | 3131 | HLF | hepatic leukemia factor (HLF), mRNA. | -1.3 | -1.1 |
| 221606_s_at | 79366 | HMGN5 | high mobility group nucleosome binding domain 5 (HMGN5), mRNA. | -1.8 | -1.1 |
| 213472_at | 3187 | HNRNPH1 | Heterogeneous nuclear ribonucleoprotein H1 (H) | -1.1 | -1.3 |
| 202638_s_at | 3383 | ICAM1 | intercellular adhesion molecule 1 (ICAM1), mRNA. | 8.2 | 6.1 |
| 201565_s_at | 3398 | ID2 | inhibitor of DNA binding 2, dominant negative helix-loop-helix protein (ID2), mRNA. | -1.0 | -2.9 |
| 201315_x_at | 10581 | IFITM2 | interferon induced transmembrane protein 2 (IFITM2), mRNA. | 4.0 | 5.2 |
| 212203_x_at | 10410 | IFITM3 | interferon induced transmembrane protein 3 (IFITM3), transcript variant 1, mRNA. | 4.6 | 5.2 |
| 203424_s_at | 3488 | IGFBP5 | insulin-like growth factor binding protein 5 (IGFBP5), mRNA. | 13.9 | 4.7 |
| 211959_at | 3488 | IGFBP5 | insulin-like growth factor binding protein 5 (IGFBP5), mRNA. | 11.9 | 3.7 |
| 203426_s_at | 3488 | IGFBP5 | insulin-like growth factor binding protein 5 (IGFBP5), mRNA. | 9.5 | 3.0 |
| 211958_at | 3488 | IGFBP5 | insulin-like growth factor binding protein 5 (IGFBP5), mRNA. | 4.6 | 3.7 |
| 210904_s_at | 3597 | IL13RA1 | interleukin 13 receptor, alpha 1 (IL13RA1), mRNA. | 2.4 | 2.1 |
| 213834_at | 440073 | IQSEC3 | IQ motif and Sec7 domain 3 | -1.1 | -1.8 |
| 203882_at | 10379 | IRF9 | interferon regulatory factor 9 (IRF9), mRNA. | 2.4 | 3.6 |
| 216261_at | 3690 | ITGB3 | Integrin, beta 3 (platelet glycoprotein IIIa, antigen CD61) | 2.3 | 2.0 |
| 210263_at | 3754 | KCNF1 | potassium voltage-gated channel, subfamily F, member 1 (KCNF1), mRNA. | 2.3 | 3.7 |
| 205303_at | 3764 | KCNJ8 | potassium inwardly-rectifying channel, subfamily J, member 8 (KCNJ8), mRNA. | 2.7 | 2.9 |
| 212794_s_at | 23325 | KIAA1033 | KIAA1033 (KIAA1033), mRNA. | -1.3 | -1.1 |
| 215936_s_at | 23325 | KIAA1033 | KIAA1033 (KIAA1033), mRNA. | -1.3 | -2.1 |
| 205235_s_at | 9585 | KIF20B | kinesin family member 20B (KIF20B), mRNA. | -1.7 | -1.1 |
| 216713_at | 889 | KRIT1 | KRIT1, ankyrin repeat containing (KRIT1), transcript variant 4, mRNA. | -1.3 | -1.2 |
| 200914_x_at | 3895 | KTN1 | kinectin 1 (kinesin receptor) (KTN1), transcript variant 4, mRNA. | -1.2 | -1.2 |
| 208949_s_at | 3958 | LGALS3 | lectin, galactoside-binding, soluble, 3 (LGALS3), transcript variant 1, mRNA. | 2.7 | 3.2 |
| 214809_at | 100294145 | LOC100294145 | Uncharacterized LOC100294145 | -1.5 | -1.3 |
| 221973_at | 100506076 | LOC100506076 | PREDICTED: hypothetical LOC100506076, transcript variant 2 (LOC100506076), miscRNA. | -1.5 | -1.2 |
| 221833_at | 100507577 | LOC100507577 | uncharacterized LOC100507577 (LOC100507577), non-coding RNA. | -1.2 | -1.2 |
| 206584_at | 23643 | LY96 | lymphocyte antigen 96 (LY96), transcript variant 1, mRNA. | 2.1 | 3.0 |
| 219188_s_at | 28992 | MACROD1 | MACRO domain containing 1 (MACROD1), mRNA. | 2.0 | 2.6 |
| 204041_at | 4129 | MAOB | Monoamine oxidase B | 6.3 | 12.6 |
| 219278_at | 9064 | MAP3K6 | mitogen-activated protein kinase kinase kinase 6 (MAP3K6), mRNA. | 2.3 | 2.4 |
| 211500_at | 5600 | MAPK11 | mitogen-activated protein kinase 11 (MAPK11), mRNA. | 2.1 | 2.3 |
| 206091_at | 4148 | MATN3 | matrilin 3 (MATN3), mRNA. | 3.7 | 2.4 |
| 209672_s_at | 54468 | MIOS | Missing oocyte, meiosis regulator, homolog (Drosophila) | -1.3 | -1.8 |
| 219703_at | 55329 | MNS1 | meiosis-specific nuclear structural 1 (MNS1), mRNA. | -1.0 | -1.1 |
| 212755_at | 23041 | MON2 | MON2 homolog (S. cerevisiae) (MON2), mRNA. | -2.4 | -1.8 |
| 37408_at | 9902 | MRC2 | mannose receptor, C type 2 (MRC2), mRNA. | 2.5 | 2.5 |
| 220346_at | 441024 | MTHFD2L | methylenetetrahydrofolate dehydrogenase (NADP+ dependent) 2-like (MTHFD2L), mRNA. | -1.1 | -1.0 |
| 218663_at | 64151 | NCAPG | non-SMC condensin I complex, subunit G (NCAPG), mRNA. | -1.2 | -1.0 |
| 218662_s_at | 64151 | NCAPG | non-SMC condensin I complex, subunit G (NCAPG), mRNA. | -1.4 | -1.2 |
| 202149_at | 4739 | NEDD9 | neural precursor cell expressed, developmentally down-regulated 9 (NEDD9), transcript variant 3, mRNA. | 2.4 | 2.4 |
| 202238_s_at | 4837 | NNMT | nicotinamide N-methyltransferase (NNMT), mRNA. | 40.2 | 157.4 |
| 202237_at | 4837 | NNMT | nicotinamide N-methyltransferase (NNMT), mRNA. | 12.2 | 47.9 |
| 209957_s_at | 4878 | NPPA | natriuretic peptide A (NPPA), mRNA. | -1.6 | -1.5 |
| 213273_at | 26011 | ODZ4 | odz, odd Oz/ten-m homolog 4 (Drosophila) (ODZ4), mRNA. | 2.2 | 4.0 |
| 202780_at | 5019 | OXCT1 | 3-oxoacid CoA transferase 1 (OXCT1), nuclear gene encoding mitochondrial protein, mRNA. | -1.4 | -1.2 |
| 202733_at | 8974 | P4HA2 | prolyl 4-hydroxylase, alpha polypeptide II (P4HA2), transcript variant 4, mRNA. | 2.3 | 7.6 |
| 211564_s_at | 8572 | PDLIM4 | PDZ and LIM domain 4 (PDLIM4), transcript variant 2, mRNA. | 2.8 | 2.5 |
| 214175_x_at | 8572 | PDLIM4 | PDZ and LIM domain 4 | 2.4 | 2.4 |
| 209581_at | 11145 | PLA2G16 | phospholipase A2, group XVI (PLA2G16), transcript variant 2, mRNA. | 2.1 | 2.1 |
| 205479_s_at | 5328 | PLAU | plasminogen activator, urokinase (PLAU), transcript variant 2, mRNA. | 5.9 | 2.3 |
| 211668_s_at | 5328 | PLAU | plasminogen activator, urokinase (PLAU), transcript variant 1, mRNA. | 5.5 | 2.0 |
| 205203_at | 5337 | PLD1 | phospholipase D1, phosphatidylcholine-specific (PLD1), transcript variant 1, mRNA. | 2.1 | 2.8 |
| 212037_at | 5411 | PNN | pinin, desmosome associated protein (PNN), mRNA. | -1.4 | -1.1 |
| 208995_s_at | 9360 | PPIG | peptidylprolyl isomerase G (cyclophilin G) (PPIG), mRNA. | -1.6 | -1.3 |
| 203407_at | 5493 | PPL | periplakin (PPL), mRNA. | 2.5 | 2.3 |
| 214917_at | 5562 | PRKAA1 | Protein kinase, AMP-activated, alpha 1 catalytic subunit | -1.5 | -1.1 |
| 218683_at | 58155 | PTBP2 | polypyrimidine tract binding protein 2 (PTBP2), mRNA. | -1.1 | -1.3 |
| 208522_s_at | 5727 | PTCH1 | patched 1 (PTCH1), transcript variant 1a', mRNA. | -1.0 | -1.1 |
| 215813_s_at | 5742 | PTGS1 | prostaglandin-endoperoxide synthase 1 (prostaglandin G/H synthase and cyclooxygenase) (PTGS1), transcript variant 2, mRNA. | 2.3 | 2.2 |
| 206574_s_at | 11156 | PTP4A3 | protein tyrosine phosphatase type IVA, member 3 (PTP4A3), transcript variant 1, mRNA. | 3.0 | 6.7 |
| 209695_at | 11156 | PTP4A3 | protein tyrosine phosphatase type IVA, member 3 (PTP4A3), transcript variant 2, mRNA. | 2.3 | 4.7 |
| 200636_s_at | 5792 | PTPRF | protein tyrosine phosphatase, receptor type, F (PTPRF), transcript variant 2, mRNA. | 2.1 | 2.2 |
| 208789_at | 284119 | PTRF | polymerase I and transcript release factor (PTRF), mRNA. | 2.7 | 3.8 |
| 208790_s_at | 284119 | PTRF | polymerase I and transcript release factor (PTRF), mRNA. | 2.5 | 2.0 |
| 221025_x_at | 83448 | PUS7L | pseudouridylate synthase 7 homolog (S. cerevisiae)-like (PUS7L), transcript variant 2, mRNA. | -1.4 | -3.4 |
| 210455_at | 27291 | R3HCC1L | R3H domain and coiled-coil containing 1-like (R3HCC1L), transcript variant 4, mRNA. | -1.2 | -1.3 |
| 205296_at | 5933 | RBL1 | Retinoblastoma-like 1 (p107) | -1.2 | -1.1 |
| 212917_x_at | 5965 | RECQL | RecQ protein-like (DNA helicase Q1-like) (RECQL), transcript variant 1, mRNA. | -1.2 | -1.0 |
| 205091_x_at | 5965 | RECQL | RecQ protein-like (DNA helicase Q1-like) (RECQL), transcript variant 1, mRNA. | -1.3 | -1.0 |
| 212646_at | 23180 | RFTN1 | raftlin, lipid raft linker 1 (RFTN1), mRNA. | 2.1 | 5.8 |
| 214700_x_at | 55183 | RIF1 | RAP1 interacting factor homolog (yeast) (RIF1), transcript variant 1, mRNA. | -1.3 | -1.5 |
| 200872_at | 6281 | S100A10 | S100 calcium binding protein A10 (S100A10), mRNA. | 2.8 | 3.5 |
| 204166_at | 22904 | SBNO2 | strawberry notch homolog 2 (Drosophila) (SBNO2), transcript variant 2, mRNA. | 2.9 | 3.3 |
| 202376_at | 12 | SERPINA3 | serpin peptidase inhibitor, clade A (alpha-1 antiproteinase, antitrypsin), member 3 (SERPINA3), mRNA. | 26.8 | 96.6 |
| 204614_at | 5055 | SERPINB2 | serpin peptidase inhibitor, clade B (ovalbumin), member 2 (SERPINB2), transcript variant 2, mRNA. | 9.0 | 5.2 |
| 202628_s_at | 5054 | SERPINE1 | serpin peptidase inhibitor, clade E (nexin, plasminogen activator inhibitor type 1), member 1 (SERPINE1), transcript variant 2, mRNA. | 7.1 | 10.6 |
| 202627_s_at | 5054 | SERPINE1 | serpin peptidase inhibitor, clade E (nexin, plasminogen activator inhibitor type 1), member 1 (SERPINE1), transcript variant 2, mRNA. | 4.4 | 3.3 |
| 200986_at | 710 | SERPING1 | serpin peptidase inhibitor, clade G (C1 inhibitor), member 1 (SERPING1), transcript variant 1, mRNA. | 8.0 | 4.3 |
| 220974_x_at | 81855 | SFXN3 | sideroflexin 3 (SFXN3), mRNA. | 2.1 | 2.6 |
| 208078_s_at | 150094 | SIK1 | salt-inducible kinase 1 (SIK1), mRNA. | 2.1 | 2.5 |
| 207074_s_at | 6570 | SLC18A1 | solute carrier family 18 (vesicular monoamine), member 1 (SLC18A1), transcript variant 1, mRNA. | 3.5 | 11.1 |
| 204981_at | 5002 | SLC22A18 | solute carrier family 22, member 18 (SLC22A18), transcript variant 1, mRNA. | 2.1 | 2.7 |
| 216236_s_at | 144195 | SLC2A14 | solute carrier family 2 (facilitated glucose transporter), member 14 (SLC2A14), mRNA. | 2.5 | 7.2 |
| 202498_s_at | 6515 | SLC2A3 | solute carrier family 2 (facilitated glucose transporter), member 3 (SLC2A3), mRNA. | 3.0 | 13.2 |
| 222088_s_at | 6515 | SLC2A3 | solute carrier family 2 (facilitated glucose transporter), member 3 (SLC2A3), mRNA. | 2.6 | 11.4 |
| 202497_x_at | 6515 | SLC2A3 | solute carrier family 2 (facilitated glucose transporter), member 3 (SLC2A3), mRNA. | 2.5 | 11.9 |
| 202499_s_at | 6515 | SLC2A3 | solute carrier family 2 (facilitated glucose transporter), member 3 (SLC2A3), mRNA. | 2.4 | 12.1 |
| 209258_s_at | 9126 | SMC3 | structural maintenance of chromosomes 3 (SMC3), mRNA. | -1.6 | -1.5 |
| 209257_s_at | 9126 | SMC3 | structural maintenance of chromosomes 3 (SMC3), mRNA. | -1.7 | -1.2 |
| 209259_s_at | 9126 | SMC3 | structural maintenance of chromosomes 3 (SMC3), mRNA. | -1.8 | -1.3 |
| 212569_at | 23347 | SMCHD1 | structural maintenance of chromosomes flexible hinge domain containing 1 (SMCHD1), mRNA. | -1.3 | -1.4 |
| 212579_at | 23347 | SMCHD1 | structural maintenance of chromosomes flexible hinge domain containing 1 (SMCHD1), mRNA. | -1.5 | -1.1 |
| 206359_at | 9021 | SOCS3 | Suppressor of cytokine signaling 3 | 2.9 | 5.3 |
| 209875_s_at | 6696 | SPP1 | secreted phosphoprotein 1 (SPP1), transcript variant 4, mRNA. | 5.9 | 4.4 |
| 204299_at | 10772 | SRSF10 | serine/arginine-rich splicing factor 10 (SRSF10), transcript variant 3, mRNA. | -1.2 | -1.1 |
| 209023_s_at | 10735 | STAG2 | stromal antigen 2 (STAG2), transcript variant 4, mRNA. | -1.2 | -1.4 |
| 208992_s_at | 6774 | STAT3 | signal transducer and activator of transcription 3 (acute-phase response factor) (STAT3), transcript variant 2, mRNA. | 2.5 | 2.1 |
| 219262_at | 79723 | SUV39H2 | suppressor of variegation 3-9 homolog 2 (Drosophila) (SUV39H2), transcript variant 4, mRNA. | -1.0 | -1.4 |
| 221618_s_at | 51616 | TAF9B | TAF9B RNA polymerase II, TATA box binding protein (TBP)-associated factor, 31kDa (TAF9B), mRNA. | -1.4 | -2.4 |
| 200916_at | 8407 | TAGLN2 | transgelin 2 (TAGLN2), mRNA. | 6.5 | 2.3 |
| 210978_s_at | 8407 | TAGLN2 | transgelin 2 (TAGLN2), mRNA. | 5.9 | 2.0 |
| 209650_s_at | 25771 | TBC1D22A | TBC1 domain family, member 22A (TBC1D22A), mRNA. | 2.9 | 4.1 |
| 204064_at | 9984 | THOC1 | THO complex 1 (THOC1), mRNA. | -1.0 | -1.0 |
| 214920_at | 221981 | THSD7A | thrombospondin, type I, domain containing 7A (THSD7A), mRNA. | -1.2 | -1.1 |
| 206555_s_at | 55623 | THUMPD1 | THUMP domain containing 1 (THUMPD1), mRNA. | -1.2 | -1.0 |
| 214581_x_at | 27242 | TNFRSF21 | Tumor necrosis factor receptor superfamily, member 21 | 2.5 | 3.3 |
| 210995_s_at | 373 | TRIM23 | tripartite motif containing 23 (TRIM23), transcript variant alpha, mRNA. | -1.2 | -1.4 |
| 204732_s_at | 373 | TRIM23 | tripartite motif containing 23 (TRIM23), transcript variant alpha, mRNA. | -1.4 | -1.2 |
| 201745_at | 5756 | TWF1 | twinfilin, actin-binding protein, homolog 1 (Drosophila) (TWF1), transcript variant 1, mRNA. | -1.1 | -1.2 |
| 202412_s_at | 7398 | USP1 | ubiquitin specific peptidase 1 (USP1), transcript variant 3, mRNA. | -1.1 | -1.5 |
| 210681_s_at | 9958 | USP15 | ubiquitin specific peptidase 15 (USP15), transcript variant 2, mRNA. | -1.1 | -1.2 |
| 210102_at | 4013 | VWA5A | von Willebrand factor A domain containing 5A (VWA5A), transcript variant 2, mRNA. | 3.1 | 7.4 |
| 201595_s_at | 55854 | ZC3H15 | zinc finger CCCH-type containing 15 (ZC3H15), mRNA. | -1.1 | -1.0 |
| 222237_s_at | 7771 | ZFP112 | zinc finger protein 112 homolog (mouse) (ZFP112), transcript variant 2, mRNA. | -1.4 | -1.2 |
| 205739_x_at | 51427 | ZNF107 | zinc finger protein 107 (ZNF107), transcript variant 2, mRNA. | -2.1 | -1.1 |
| 219495_s_at | 7733 | ZNF180 | zinc finger protein 180 (ZNF180), mRNA. | -1.1 | -1.1 |
| 220350_at | 9310 | ZNF235 | zinc finger protein 235 (ZNF235), mRNA. | -1.2 | -1.3 |
| 219540_at | 10308 | ZNF267 | zinc finger protein 267 (ZNF267), transcript variant 2, mRNA. | -1.8 | -1.2 |
| 207190_at | 23140 | ZZEF1 | Zinc finger, ZZ-type with EF-hand domain 1 | 3.7 | 4.1 |
