# Supplementary material for: Coculture with astrocytes reduces the radiosensitivity of glioblastoma stem-like cells and identifies additional targets for radiosensitization
Source: Cancer Med. 2015 Oct 30;4(11):1705–16. doi: 10.1002/cam4.510 (PMC4673998; doi:10.1002/cam4.510)
Supplement: Supplementary file 7 [file cam40004-1705-sd7.docx]

**Table S3: Top 10 upstream regulators of the 112 transcripts whose expression was increased (≥2-fold, p ≤ 0.05) in co-cultured GSCs compared to mono-cultures.**

|  | ***Upstream Regulator*** | ***Molecule Type*** | ***Predicted Activation State*** | | ***Activation z-score*** | ***# target molecules*** |
| --- | --- | --- | --- | --- | --- | --- |
|  | IL6 | cytokine | Activated | | 4.534 | 26 |
|  | IL1B | cytokine | Activated | | 4.009 | 25 |
|  | IGF1 | growth factor | Activated | | 3.794 | 17 |
|  | IFNG | cytokine | Activated | | 3.780 | 31 |
|  | TNF | cytokine | Activated | | 3.749 | 35 |
|  | NFkB | complex | Activated | | 3.653 | 24 |
|  | IL1 | group | Activated | | 3.634 | 14 |
|  | STAT3 | transcription regulator | | Activated | 3.542 | 15 |
|  | AGT | growth factor | Activated | | 3.497 | 14 |
|  | PDFG BB | complex | Activated | | 3.441 | 16 |
